# Microglia P2Y_13_ Receptors Prevent Astrocyte Proliferation Mediated by P2Y_1_ Receptors

**DOI:** 10.3389/fphar.2018.00418

**Published:** 2018-05-03

**Authors:** Clara Quintas, Nuno Vale, Jorge Gonçalves, Glória Queiroz

**Affiliations:** ^1^Laboratory of Pharmacology, Department of Drug Sciences, Faculty of Pharmacy, University of Porto, Porto, Portugal; ^2^REQUIMTE/LAQV, University of Porto, Porto, Portugal; ^3^REQUIMTE/UCIBIO, University of Porto, Porto, Portugal; ^4^MedInUP, University of Porto, Porto, Portugal

**Keywords:** P2Y_13_ receptors, P2Y_1_ receptors, microglia, cell proliferation, astrocyte-microglia communication, IL-1β, anti-IL-1α, anti-TNF-α

## Abstract

Cerebral inflammation is a common feature of several neurodegenerative diseases that requires a fine interplay between astrocytes and microglia to acquire appropriate phenotypes for an efficient response to neuronal damage. During brain inflammation, ATP is massively released into the extracellular medium and converted into ADP. Both nucleotides acting on P2 receptors, modulate astrogliosis through mechanisms involving microglia-astrocytes communication. In previous studies, primary cultures of astrocytes and co-cultures of astrocytes and microglia were used to investigate the influence of microglia on astroglial proliferation induced by ADPβS, a stable ADP analog. In astrocyte cultures, ADPβS increased cell proliferation through activation of P2Y_1_ and P2Y_12_ receptors, an effect abolished in co-cultures (of astrocytes with ∼12.5% microglia). The possibility that the loss of the ADPβS-mediated effect could have been caused by a microglia-induced degradation of ADPβS or by a preferential microglial localization of P2Y_1_ or P2Y_12_ receptors was excluded. Since ADPβS also activates P2Y_13_ receptors, the contribution of microglial P2Y_13_ receptors to prevent the proliferative effect of ADPβS in co-cultures was investigated. The results obtained indicate that P2Y_13_ receptors are low expressed in astrocytes and mainly expressed in microglia. Furthermore, in co-cultures, ADPβS induced astroglial proliferation in the presence of the selective P2Y_13_ antagonist MRS 2211 (3 μM) and of the selective P2Y_12_ antagonist AR-C66096 (0.1 μM), suggesting that activation of microglial P2Y_12_ and P2Y_13_ receptors may induce the release of messengers that inhibit astroglial proliferation mediated by P2Y_1,12_ receptors. In this microglia-astrocyte paracrine communication, P2Y_12_ receptors exert opposite effects in astroglial proliferation as a result of its cellular localization: cooperating in astrocytes with P2Y_1_ receptors to directly stimulate proliferation and in microglia with P2Y_13_ receptors to prevent proliferation. IL-1β also attenuated the proliferative effect of ADPβS in astrocyte cultures. However, in co-cultures, the anti-IL-1β antibody was unable to recover the ADPβS-proliferative effect, an effect that was achieved by the anti-IL-1α and anti-TNF-α antibodies. It is concluded that microglia control the P2Y_1,12_ receptor-mediated astroglial proliferation through a P2Y_12,13_ receptor-mediated mechanism alternative to the IL-1β suppressive pathway that may involve the contribution of the cytokines IL-1α and TNF-α.

## Introduction

Astrocytes and microglia respond to all types of central nervous system (CNS) insults, undergoing through several morphological and functional changes to adapt to the requirements of the surrounding inflammatory response. Microglia initiate a response that starts with their activation and with a rapid mobilization to the site of injury, to phagocyte death cells and to remove cell debris (Hanisch and Kettenmann, 2007). Astrocytes also become reactive, coursing with glial fibrillary acidic protein up-regulation, hypertrophy and, in some cases, proliferation, to form a glial scar that limits the damaged area and prevents widespread inflammation (Sofroniew, 2014).

Pro-inflammatory cytokines attain high extracellular concentrations at the early stages of the inflammatory response and trigger, or modulate, the course of astrogliosis (Buffo et al., 2010). Since microglia are the main source of inflammatory mediators, these cells are regarded as active players in orchestrating the progression of astrogliosis. Microglia activation, and an increase in microglia-derived mediators, are primary events that occur in the inflammatory response, even before the astrocytes response (Zhang et al., 2010), supporting the relevance of a continuous communication between microglia and astrocytes during inflammatory insults, to control astrogliosis.

Purinergic signaling plays a central role in the microglia-astrocyte communication during the CNS inflammatory response. Nucleotides, leaked from dying or damaged cells, act as damage-associated molecular patterns, signaling the damage and triggering different actions through activation of ionotropic P2X or metabotropic P2Y receptors, expressed in astrocytes and microglia (Di Virgilio et al., 2009). For example, ATP and its metabolite ADP, activate microglia P2Y_1_, P2Y_12_ and P2X_4_ receptors (Ohsawa et al., 2007; De Simone et al., 2010) that act as sensors to guide microglia to the site where the inflammatory response is occurring (Davalos et al., 2005). In astrocytes, activation of P2Y_1_ and P2Y_12_ receptors causes hypertrophy and stimulates proliferation, to create the glial scar that confines the lesion site, restraining the secondary neuronal damage (Franke et al., 2004; Quintas et al., 2011a).

It is also known that inflammatory mediators, released by microglia, modulate the expression and function of G protein-coupled receptors that induce an increase in [Ca^2+^]_i_ in astrocytes (Hamby et al., 2012). P2Y_1_ receptors are metabotropic receptors which, in astrocytes, mediate an increase in [Ca^2+^]_i_ (Fischer et al., 2009) and trigger astroglial proliferation, an hallmark of astrogliosis (Franke et al., 2004). Therefore, it is expectable that microglia inflammatory mediators may modulate P2Y_1_ receptors activity and consequently, influence astrogliosis progression.

We have previously confirmed that astroglial proliferation may be induced not only by activation of astrocyte P2Y_1_ receptors, but also by P2Y_12_ receptors (Quintas et al., 2011a). It was further demonstrated that P2Y_1,12_ receptor-mediated astroglial proliferation is inhibited in co-cultures of astrocytes and microglia, when microglia P2Y receptors are also activated (Quintas et al., 2011b). In those studies, it was excluded the possibility that the loss of the ADPβS-mediated effect could have been caused by a microglia-induced metabolisation of the compound or by a preferential microglial localization of P2Y_1_ or P2Y_12_ receptors, but it was evident that microglial P2Y receptors induced the release of diffusible paracrine mediator(s) to prevent ADPβS-mediated astroglial proliferation. Since ADPβS also activates P2Y_13_ receptors, this subtype arise as a promising candidate to mediate this microglia-astrocytes communication.

Expression of P2Y_13_ receptors was previously detected in microglia from the whole brain of mice (Crain et al., 2009), but also in astrocytes of several brain regions (Fumagalli et al., 2004; Carrasquero et al., 2005; Fischer et al., 2009), and functional studies support the relevance of these receptor subtypes in both types of cells. P2Y_13_ receptors activation was shown to elicit [Ca^2+^]_i_ increase in microglia (Zeng et al., 2014) and astrocytes (Carrasquero et al., 2009; Fischer et al., 2009) and to mediate the release of several pro-inflammatory cytokines by microglia, such as IL-1β, IL-6 and TNF-α (Liu et al., 2017).

In the previous studies, P2Y receptor-mediated astrocyte proliferation was induced by ADPβS, a stable ADP analog selective for P2Y_1_, P2Y_12_ and P2Y_13_ receptors (Quintas et al., 2011a). However, the mechanisms behind this P2Y receptor-mediated communication between microglia and astrocytes are still largely unknown, namely the subtype of receptors involved and the identity of such mediator(s). Therefore, the aim of the present study was to clarify the role of each of these P2Y receptor subtypes in the microglia modulation of astroglial proliferation.

It is concluded that microglial P2Y_12_ and P2Y_13_ are the receptor subtypes involved in preventing astroglial proliferation mediated by ADPβS in co-cultures of astrocytes and microglia. As far as putative mediator(s) are concerned, the present study further shows that, in spite of all indications that microglia-derived IL-1β could be a strong candidate to prevent ADPβS-induced astroglial proliferation, the microglial P2Y receptor inhibition of astrocyte proliferation occurs through a IL-1β independent mechanism, which involves the release of IL-1α and TNF-α.

## Materials and Methods

### Drugs and Antibodies

The following antibodies and drugs were used: goat anti-mouse IgG conjugated to Alexa Fluor 488 from Invitrogen (Barcelona, Spain); rabbit polyclonal anti-P2Y_13_ from Alomone Laboratories (Jerusalem, Israel); mouse monoclonal anti-CD11b, and goat anti-rabbit IgG conjugated to horseradish peroxidase from Santa Cruz Biotechnology (Santa Cruz, CA, United States); 5-bromo-20-deoxyuridine (BrdU), rabbit polyclonal anti-BrdU, rabbit polyclonal anti-α tubulin, goat anti-rabbit IgG conjugated to Alexa Fluor 594 from Abcam (Cambridge, United Kingdom); rabbit and mouse anti-glial fibrillary acidic protein (anti-GFAP), recombinant rat interleukin-1β, rabbit polyclonal anti-interleukin-1β antibody, adenosine 5’-*O*-(3-thio)-diphosphate tetralithium (ADPβS), 2′-(4-hydroxyphenyl)-5-(4-methyl-1-piperazinyl)-2,5′-bi-1H-benzimidazole trihydrochloride hydrate(Hoechst 33258), penicillin and streptomycin from Sigma-Aldrich (Sintra, Portugal); 2-(propylthio)adenosine-5′-*O*-(β,γ-difluoromethylene)triphosphate tetrasodium (AR-C66096), 2-[(2-chloro-5-nitrophenyl) azo]-5-hydroxy-6-methyl-3-[(phos-phonooxy)methyl]-4-pyridinecarboxaldehyde disodium (MRS 2211) and (1R^∗^,2S^∗^)-4-[2-Iodo-6-(methylamino)-9H-purin-9-yl]-2-(phosphonooxy)bicyclo[3.1.0]hexane-1-methanol dihydro-gen phosphate ester tetraammonium (MRS 2500) from Tocris (Bristol, United Kingdom); *methyl*-[^3^H]-thymidine (specific activity 80–86 Ci.mmol^-1^) and enhanced chemiluminescence (ECL) Western blotting system from Amersham Biosciences (Lisbon, Portugal); goat polyclonal IL-1α antibody, mouse polyclonal TNF-α antibody from ThermoFisher Scientific (Lisbon, Portugal). Stock solutions of drugs were prepared with distilled water and kept at -20°C. Solutions of drugs were prepared from stock solutions diluted in culture medium immediately before use.

### Cell Cultures

Animal handling and experiments were conducted according to the guidelines of the Directive 2010/63/EU of the European Parliament and the Council of the European Union and the Organismo Responsável pelo Bem-Estar Animal (ORBEA) from ICBAS-UP. Primary cortical astroglial cultures were prepared from offspring of Wistar rats (Charles River, Barcelona, Spain) as previously described (McCarthy and de Vellis, 1980). Briefly, the brains were placed in ice-cold Dulbecco’s phosphate buffered calcium-free saline solution (DPBS) containing 0.2% glucose. The meninges and blood vessels were removed from hemispheres and after washing twice with ice-cold DPBS, they were cut into small pieces in culture medium, i.e., Dulbecco’s modified Eagle medium containing 3.7 g/L NaHCO_3_, 1.0 g/L D-glucose and stable glutamine, supplemented with 50 U/ml penicillin, 50 μg/ml streptomycin. Tissue from two hemispheres was dissociated by triturating in 10 ml culture medium. The cell suspension obtained was passed through a 40-μm pore nylon mesh and then centrifuged at 200 × *g* for 5 min and the supernatant discharged. Centrifugation followed by cell suspension was repeated twice and the pellet obtained was suspended in culture medium supplemented with 10% foetal bovine serum (FBS), and seeded at a density of 2 × 10^5^cells/ml. Cultures were incubated at 37°C in a humidified atmosphere of 95% air, 5% CO_2_ and the medium was replaced 1 day after preparation and subsequently twice a week. Confluent co-cultures of astrocytes and microglia were obtained at DIV14-18.

To prepare highly enriched astroglial cultures, that were named astrocytes cultures, confluent co-cultures were shaken overnight at 200 rpm to detach microglia sitting on the top of the astroglial monolayer and then trypsinized and subcultured to remove microglia trapped within the astroglial monolayer (Saura, 2007).

The suppernant obtained from confluent co-cultures after shaken overnight, which was enriched in microglia, was not discharged being used to prepared microglia cultures as previously described (Ni and Aschner, 2010; Deierborg, 2013). Briefly, the suppernant of shaken co-cultures was collect in 50 ml tubes previously cooled to 4°C and centrifuged at 1000 rpm for 10 min at 4°C. The supernant was discarded, the pellet obtained was resuspended in complete medium and cells were seeded at a density of 10^6^ cells/ml. The surface of the supports used for culturing micoglia were previously coated with poly-L-lysine for better cell adhesion. To promote selective adhesion of microglia, culture medium was changed 1 h after seeding and replaced by complete medium containing 5 ng/ml M-CSF to promote microglial growth.

Co-cultures were used in experiments at DIV23. Highly enriched astrocyte cultures and microglia cultures were used at DIV6 after purification. All types of cultures were synchronized to a quiescent phase of the cell cycle, by shifting serum concentration to 0.1% FBS for 48 h before performing the experiments.

### DNA Synthesis

At DIV23, cultures grown in 24-well plates, were incubated with ADPβS, IL-1β, or solvent for 48 h and methyl-[^3^H]-thymidine was added to the medium in the last 24 h, at a concentration of 1 μCi/ml. When present, antagonists were added to the medium 1 h before ADPβS. IL-1β and the anti-ILs antibodies tested were added at the same time as ADPβS. At the end of the 48 h period of incubation, cells were rinsed with PBS, fixed with 10% of trichloroacetic acid for 30 min at 4°C, washed with ice-cold 5% trichloroacetic acid and rinsed again with PBS. Protein content and methyl-[^3^H]-thymidine incorporation were evaluated after cell lysis with 0.2 M NaOH. The effect of drugs in cell proliferation was determined by *methyl*-[^3^H]-thymidine incorporation, quantified by liquid scintillation spectrometry (Beckman LS 6500, Beckman Instruments, Fullerton, CA, United States) and normalized by the protein content determined by the Bradford method.

### Immunocytochemistry

Cell cultures grown on 13 mm round coverslips were fixed in 4% paraformaldehyde and 4% sucrose in phosphate buffered saline (PBS; 100 mM NaH_2_PO_4_, 50 mM NaCl, pH adjusted to 7.3) and then incubated with 10% FBS, 1% bovine serum albumin, 0.1% Triton X, 0.05% NaN3 in PBS for 1 h. For double labeling astrocytes and microglia, and for P2Y_13_ receptors localization, cultures were incubated overnight at 4°C with the following primary antibodies, diluted in 5% FBS, 1% bovine serum albumin, 0.1% Triton X, 0.05% NaN3 in PBS: rabbit or mouse anti-glial fibrillary acidic protein (anti-GFAP, 1:600), mouse anti-CD11b (1:50) and rabbit anti-P2Y_13_ (1:200). Visualization of GFAP, CD11b and P2Y_13_ receptors positive cells was accomplished upon 1 h incubation, at room temperature, with the secondary antibodies anti-rabbit IgG conjugated to Alexa Fluor 594 and anti-mouse IgG conjugated to Alexa Fluor 488 (both at 1:400). In negative controls, the primary antibody was omitted. Cell nuclei were labeled with Hoechst 33258 (5 μg/ml) for 1 min at room temperature. To evaluate the percentage of microglia, the two types of cultures were processed in parallel and about 200 cells were counted in each culture. The number of CD11b positive cells was expressed as percentage of the total number of cells counted. Images were captured with Lionheart^TM^ FX Automated Microscope (Biotek, United Kingdom).

### BrdU Staining

In astrocyte cultures, proliferation of astrocytes or contaminating microglia was identified through double labeling of GFAP or CD11b and 5-bromo-20-deoxyuridine (BrdU) positive cells. Astrocytes cultures grown on 13 mm round coverslips were incubated with ADPβS 300 μM for 48 h. BrdU (100 μM) was added to the medium for the last 24 h, after which time the cells were incubated with mouse anti-CD11b (1:50) for 30 min at 37°C and then fixed with in 4% paraformaldehyde and 4% sucrose in PBS for 15 min at room temperature. Coverslips were washed with PBS and then incubated for 20 min with methanol to permeabilize the membranes. The BrdU epitope was exposed by incubating the cells in 2 M hydrochloric acid for 1 h at 37°C followed by neutralization with 0.1 M sodium borate, pH 8.5 for 20 min. Cell cultures were blocked with 3% FBS and then incubated with mouse anti-GFAP (1:600) and rabbit anti-BrdU (1:300) for 1 h, at room temperature. Visualization of GFAP or CD11b and BrdU positive cells was accomplished upon 1 h incubation, at room temperature, with the secondary antibodies anti-rabbit IgG conjugated to Alexa Fluor 594 and anti-mouse IgG conjugated to Alexa Fluor 488 (both at 1:400). In negative controls, the primary antibody was omitted. Images were captured with Lionheart^TM^ FX Automated Microscope (Biotek, United Kingdom).

### Real Time RT-qPCR Analysis

RNA was extracted from astrocyte and microglia cultures with the RNeasy Mini Kit (QIAGEN), according to manufacturer’s instructions. RNA purity and concentration was confirmed using a Synergy HT spectrophotometer (Biotek, United Kingdom). Nine hundred ng of RNA (astrocytes) or 70 ng of RNA (microglia) were used as a template for reverse-transcriptase reactions using the NZY First-Strand cDNA Synthesis kit (NZYTech). The primer sequences, listed in **Table 1**, were designed and evaluated with Beacon Designer^TM^ Software 7 (PREMIER Biosoft). Primer specificity was assessed through NCBI BLAST analysis prior to use and, for each sample following PCR, it was verified that the dissociation curve had a single peak with an observed Tm consistent with the amplicon length. Standard dilutions of the cDNA were used to check the relative efficiency and quality of primers. Negative controls (no template cDNA) were included in all qPCR.

**Table 1:** Primer sequence table.

Gene	Forward primer	Reverse primer
P2Y_1_	5′-CTGATCTTGGGCTGTTATGG-3′	5′-GCTGTTGAGACTTGCTAGAC-3′
P2Y_12_	5′-TGTTCCTGCTGTCACTGCCTAA-3′	5′-CTCGTGCCAGACCAGACCAA-3′
P2Y_13_	5′-TGCACTTTCTCATCCGTGGT-3′	5′-GGCAGGGAGATGAGGAACAT-3′
β-actin	5′-CTGTGCTATGTTGCCCTA-3′	5′-CCGATAGTGATGACCTGAC-3′
GAPDH	5′-TTCAACGGCACAGTCAAG-3′	5′-TACTCAGCACCAGCATCA-3′

qPCR amplifications were performed in duplicate, using 0.125 μM of each primer,10 μl of 2X iTaqTM Universal SYBR Green Supermix (Bio-Rad) and 1 μl of template cDNA. qPCRs were carried out on a CFX96 Touch^TM^ Real-Time PCR Detection System (Bio-Rad) and conditions were as follows: 95°C for 3 min followed by 40 cycles of denaturation at 95°C for 10 s, 60°C annealing temperature for 30 s. Melting curves of the PCR amplicons were then generated with temperatures ranging from 55°C to 95°C, with increments of 0.5°C at a rate of 10 s/step. The melting curve data were analyzed with the CFX Manager^TM^ (ver. 2.0, Bio-Rad). The data obtained were analyzed using the method described by Pfaffl (2001). Ct values from duplicate measurements were averaged, and relative expression levels were determined by the 2^-ΔC_T_^ method. While PCRs were run to 40 cycles, all detected genes had Ct values below 31 in all of the samples examined. For each analysis GAPDH and β-actin were used for normalization.

### Western Blot Analysis

Cell cultures were rinsed with ice-cold PBS and total cell protein extracted in lysis buffer with protease inhibitors (1 mM Na_3_VO_4_, 1 mM NaF, 1 mM PMSF, 2 μg/ml aprotinin and 2 μg/ml leupeptin). Ceramic beads with 1.4 mm were added to the samples, which were disrupted with two cycles of 15 s at 5800 rpm in the Precellys Evolution homogenizer (Bertin Instruments, France). The lysate was incubated on ice for 1 h and then centrifuged at 20,000 × *g* for 45 min at 4°C. The protein concentration was determined in the supernatant and equal amounts of protein (50 μg) were boiled at 70°C for 10 min in 6x sample buffer (0.35 M Tris–HCl at pH 6.8, 10% SDS, 30% glycerol, 9.3% dithiothreitol, 0.01% bromphenol blue, 5% mercaptoethanol) and subjected to 12% SDS-PAGE (SDS-polyacrylamide gel electrophoresis). Proteins were electrotransferred onto nitrocellulose membranes overnight at 40 V in transfer buffer. Membranes were blocked at room temperature for 2 h with 5% of bovine serum albumin in PBS, and then probed for 2 h at room temperature with primary polyclonal antibody rabbit anti-P2Y_13_ (1:200) followed by the secondary antibody goat anti-rabbit IgG conjugated to horseradish peroxidase (1:10,000). Immunoblots were then stripped by incubation in stripping buffer (62.5 mM Tris-HCl, 100 mM 2-mercaptoethanol and 2% SDS, pH adjusted to 6.8) for 15 min at 50°C and blocked overnight with 5% of bovine serum albumin in PBS. Subsequently, membranes were re-probed with the primary polyclonal antibody rabbit anti-atubulin (1:1000) for 1 h at room temperature, followed by the secondary antibody. Immunocomplexes were detected using Novex ECL Chemiluminescent kit (Life Technologies) and ChemiDoc MP Imaging System (Bio-Rad, Portugal).

### Statistical Analysis

Data are expressed as means ± standard errors of the mean (S.E.M.) from n independent cell cultures tested in triplicated, or duplicated in qRT-PCR experiments. Statistical analysis was carried out using the unpaired Student’s *t*-test or one-way analysis of variance (ANOVA) followed by Dunnett’s multiple comparison test. Statistical analysis performed on 2^-ΔC_T_^ data was carried out using one-way ANOVA followed by Bonferroni’s *post hoc* comparisons tests. *P*-values lower than 0.05 were considered to indicate significant differences.

## Results

### Characterization of Glial Cultures

Two types of cell cultures were prepared, astrocytes being the predominant cell type. When no treatment was applied, cultures could be described as a monolayer of astrocytes containing a significant percentage of microglia 12.5 ± 0.2% (*n* = 4). Cultures obtained under these conditions were named astrocyte-microglia co-cultures or, more briefly, co-cultures. Confluent astrocyte-microglia co-cultures were treated to eliminate microglia (see section “Materials and Methods”), resulting in cell cultures of astrocytes with much less microglia (1.6 ± 0.1%; *n* = 4). Cultures obtained under these conditions were named astrocyte cultures. Both types of cultures, co-cultures and astrocyte cultures, were used in the experiments to identify microglia P2Y receptor subtype(s) and to explore potential paracrine mechanisms involved in the control of the ADPβS-induced astroglial proliferation.

### Glial P2Y Receptor Subtype(s) Involved in the Modulation of ADPβS-Induced Astroglial Proliferation

The proliferative effect of ADPβS was compared in astrocyte cultures and in co-cultures to clarify the influence of microglia in the astrocyte proliferation elicited by this more stable ADP analog, selective for P2Y_1_, P2Y_12_ and P2Y_13_ receptors.

**FIGURE 1:**
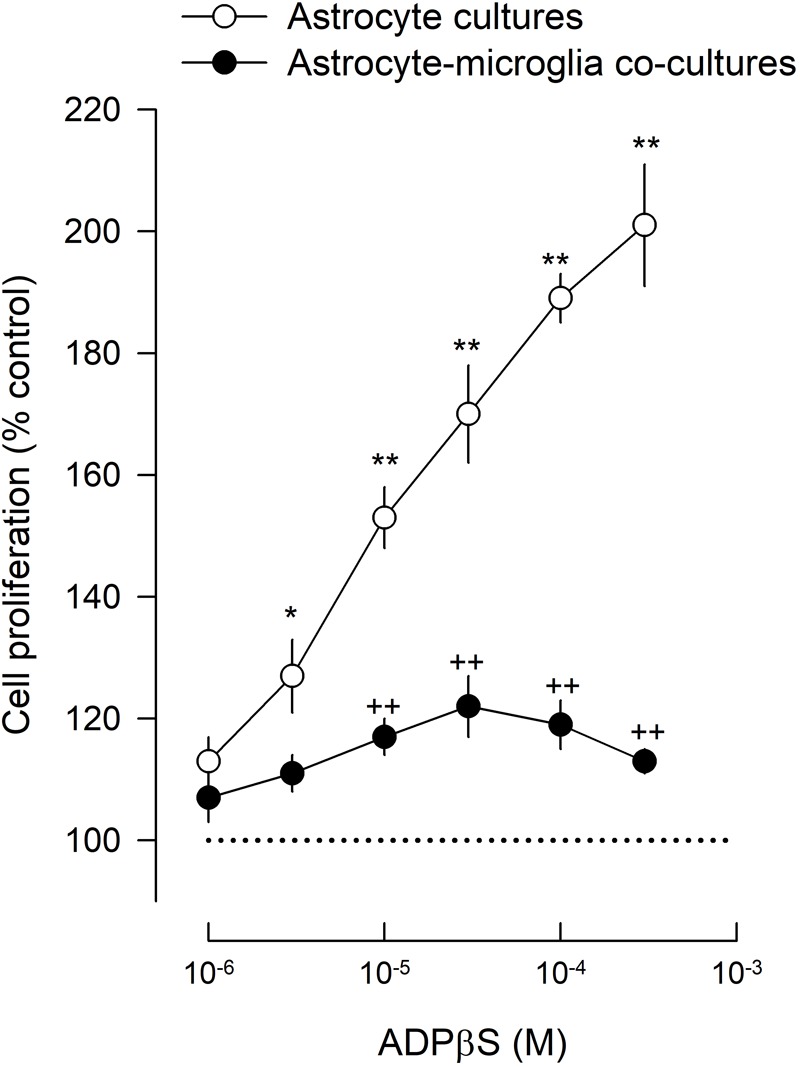
Astroglial proliferation in astrocyte cultures and astrocyte-microglia co-cultures induced by ADPβS. Cultures were incubated with ADPβS or solvent for 48 h and *methyl*-[^3^H]-thymidine (1 μCi/ml) was added in the last 24 h. Cell proliferation was estimated by *methyl*-[^3^H]-thymidine incorporation and expressed in percentage of control. Values are means ± SEM from 4 different cultures. ^∗^*P* < 0.05 and ^∗∗^*P* < 0.01, significant differences from control (solvent). ^++^*P* < 0.01, significant differences from the ADPβS in astrocyte cultures.

**FIGURE 2:**
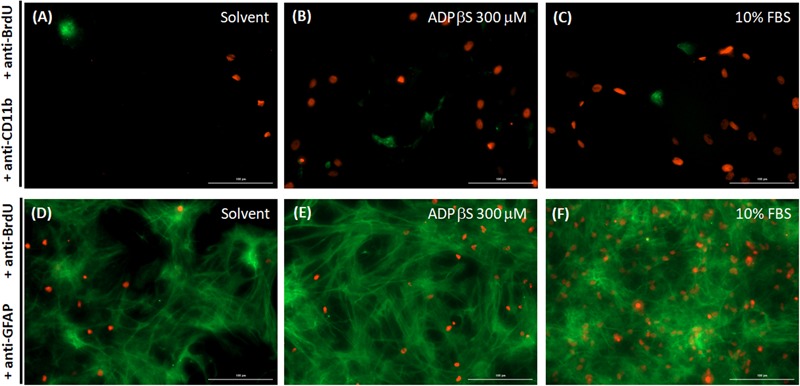
BrdU staining and its cellular localization in astrocyte cultures. Astrocyte cultures were incubated with solvent **(A,D)**, ADPβS **(B,E)** or 10% FBS, a positive control for cell proliferation **(C,F)**, for 48 h. BrdU (100 μM) was added to the medium for the last 24 h and then stained with rabbit anti-BrdU (Alexa Fluor 594, red) to visualize BrdU incorporation. Microglia were then co-labeled with mouse anti-Cd11b (**A–C**; Alexa Fluor 488, green) and astrocytes, with mouse anti-GFAP (**D–F**; Alexa Fluor 488, green). BrdU positive nuclei co-localize mainly with astrocytes, showing they are the main proliferating cells within the astrocyte cultures. Representative images from 3 different cultures. Scale bar: 100 μm.

In astrocyte cultures, ADPβS (1–300 μM), increased astroglial proliferation in a concentration-dependent manner, up to 201 ± 10% (*n* = 4; **Figure 1**). Although astroglial cultures had about 2% of contaminating microglia (see above), proliferation of astrocytes and microglia was differentiated through a double labeling of GFAP or CD11b with 5-bromo-20-deoxyuridine (BrdU), to identify the type of BrdU positive cells. The results indicate that the main proliferating cells, the BrdU positive cells, were astrocytes (**Figure 2**).

**FIGURE 3:**
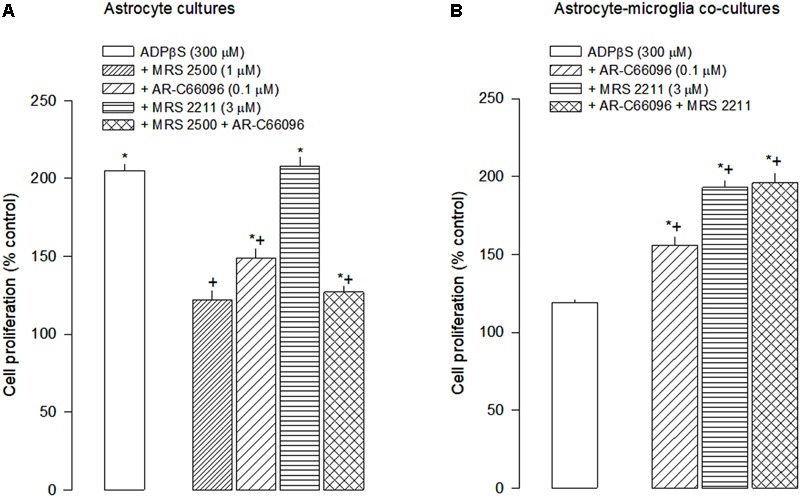
Pharmacological characterization of P2Y receptors involved in the modulation of ADPβS-mediated astroglial proliferation. **(A)** Astrocyte cultures and **(B)** astrocyte-microglia co-cultures were incubated with ADPβS or solvent for 48 h and *methyl*-[^3^H]-thymidine (1 μCi/mL) was added in the last 24 h. The P2Y antagonists: MRS 2500 (anti-P2Y_1_R), AR-C66096 (anti-P2Y_12_R) and MRS 2211 (anti-P2Y_13_R) were added to the medium 1 h before ADPβS. Cell proliferation was estimated by *methyl*-[^3^H]-thymidine incorporation and expressed in percentage of control. Values are means ± SEM from 6 to 10 different cultures. ^∗^*P* < 0.05, significant differences from control (solvent).^+^*P* < 0.05, significant differences from ADPβS alone.

The proliferative effect caused by ADPβS (300 μM) was antagonized by MRS 2500 (1 μM), a selective antagonist of P2Y_1_ receptor, by AR-C66096 (0.1 μM), a selective antagonist of P2Y_12_ receptor, but not by MRS 2211 (3 μM), a selective antagonist of P2Y_13_ receptor (**Figure 2A**). No additive antagonism was observed when both MRS 2500 (1 μM) and AR-C66096 (0.1 μM) were tested simultaneously (**Figure 3A**).

In co-cultures, ADPβS failed to cause astroglial proliferation. However, the ADPβS-induced astroglial proliferation was almost restored in the presence of MRS 2211 (3 μM), reaching proliferation levels similar to those observed in astrocyte cultures, and only partially restored by AR-C66096 (0.1 μM). When P2Y_12_ and P2Y_13_ receptors were blocked simultaneously, ADPβS proliferative effect was similar to that observed in the presence of the P2Y_13_ antagonist alone (**Figure 3B**).

### Expression and Cellular Localization of P2Y_13_ in Glial Cultures

P2Y_1_ and P2Y_12_ receptor subtypes are known to be expressed both in astrocytes (Fumagalli et al., 2003; Franke et al., 2004; Amadio et al., 2010) and microglia (Bianco et al., 2005; Ohsawa et al., 2007; De Simone et al., 2010) and expression levels of these receptor subtypes was found to be similar in both types of cultures, without any predominant cell-type localization (Quintas et al., 2011b). Therefore, considering these observations, and the results from the pharmacological approach, it remains to be explored a putative role for P2Y_13_ receptor as candidate to mediate the loss of ADPβS-induced astroglial proliferation in co-cultures. Therefore, the expression of P2Y_13_ receptors was further analyzed in both types of cultures and their cellular localization was characterized in co-cultures.

**FIGURE 4:**
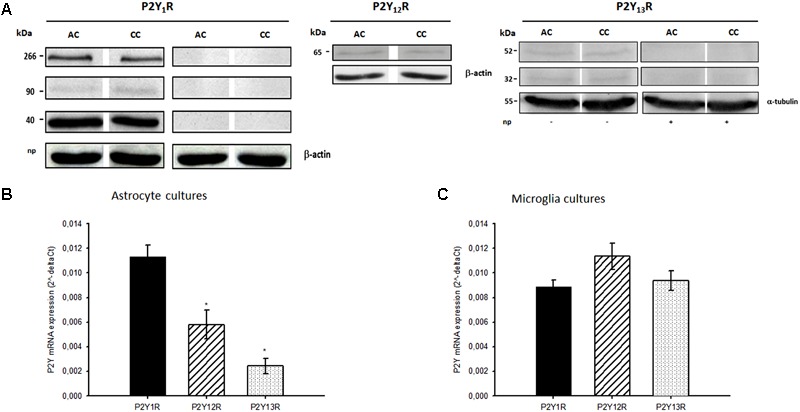
Expression of P2Y_1_, P2Y_12_ and P2Y_13_ receptors. **(A)** Representative Western blots of P2Y_1_, P2Y_12_ and P2Y_13_ receptors (P2Y_1_R, P2Y_12_R and P2Y_13_R) expression in astrocyte-microglia co-cultures (CC) and astrocyte cultures (AC). P2Y_1_R, P2Y_12_R and β-actin (43 kDa) expression data previously published by Quintas et al. (2011a,b). P2Y_13_ receptors (P2Y_13_R) and α-tubulin (55 kDa) expression were obtained from whole cell lysates. Two immunoreactive bands of 32 and 52 kDa specifically reacted with rabbit anti-P2Y_13_ antibody. These bands were absent in the presence of the respective neutralising peptide (np). mRNA expression of P2YR in **(B)** astrocyte cultures and **(C)** microglia cultures. mRNA P2YR expression were determined using qRT-PCR and normalized to GAPDH (astrocytes cultures) or β-actin (microglial). Values are means ± SEM from 3 different cultures. ^∗^*P* < 0.05, significant differences from P2Y_1_R expression.

In western blot assays, P2Y_13_ receptors expression was evidenced by two immunoreactive bands of 32 and 52 kDa that reacted with the anti-P2Y_13_ antibody. These bands were absent in the presence of the P2Y_13_ neutralizing peptide, indicating they represent specific epitopes for the anti-P2Y_13_ receptor antibody (**Figure 4A**, see also Supplementary Material). qRT-PCR experiments confirm the expression of the three receptor subtypes in astrocyte cultures, despite the significant lower expression of P2Y_12_ and P2Y_13_ receptors, when compared to that of P2Y_1_ receptors (**Figure 4B**). In contrast, the expression of these receptor subtypes was similar in microglia cultures (**Figure 4C**). The results obtained indicate that, even though at low expression levels, P2Y_13_ receptors are present in co-cultures and astrocyte cultures and that both cell types, astrocytes and microglia, express the receptor.

**FIGURE 5:**
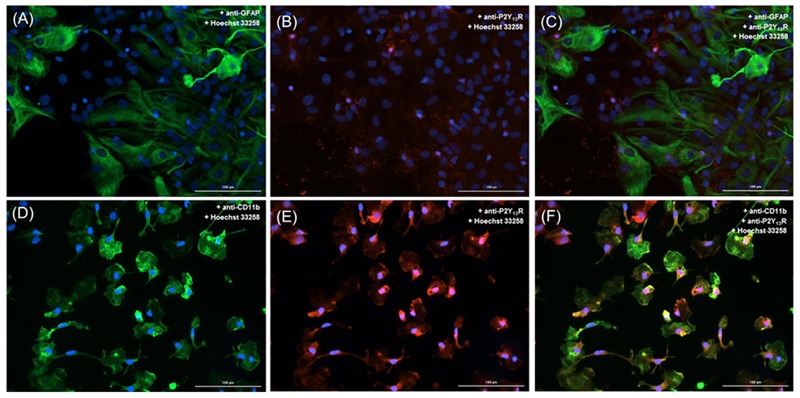
Cellular distribution and localization of P2Y_13_ receptors in astrocyte-microglia co-cultures. Astrocytes were double labeled with **(A)** mouse anti-GFAP (Alexa Fluor 488, green) and with **(B)** rabbit anti-P2Y_13_ receptor (Alexa Fluor 594, red). Microglia were double labeled with **(D)** mouse anti-CD11b (Alexa Fluor 488, green) and with **(E)** rabbit anti-P2Y_13_ receptor (Alexa Fluor 594, red). Nuclei were labeled with Hoechst 33258 (blue). P2Y_13_ receptors (red) co-localize with astrocytes **(C)** but mainly with microglia **(F)**. Scale bar: 100 μm.

Immunocytochemistry analysis of co-cultures, revealed low immunoreactivity for P2Y_13_ receptors in astrocytes (**Figures 5A–C**), and a preferentially localization in microglial cells (P2Y_13_ receptor subtype in red and the CD11b integrin in green; **Figures 5D–F**). Although astrocytes express P2Y_13_ receptors, it is clear that P2Y_13_ receptors are mostly localized in microglia, and thus, may have a more relevant role in controlling the ADPβS-induced astroglial proliferation through P2Y_1,12_ receptors.

### On the Microglia Paracrine Mediator That Prevents the ADPβS-Induced Astroglial Proliferation

Previous studies have shown that, in co-cultures, ADPβS activates microglia P2Y receptors, inducing release of non-identified diffusible messenger(s) that attenuated its proliferative effect in astrocytes (Quintas et al., 2011b). Interleukins are potential candidates, since recently it was demonstrated that activation of microglia P2Y_12,13_ receptors induces the release of IL-1β, TNF-α and IL-6 (Liu et al., 2017). Furthermore, IL-1β has been shown to decrease the activity of P2Y_1_ receptors (Scemes, 2008). In preliminary experiments, the presence of IL-1β was detected by ELISA in the supernatant of co-cultures treated with ADPβS (not shown). Therefore, it was hypothesized that activation of microglial P2Y_12,13_ receptors by ADPβS may induce release of IL-1β from microglia, which in turn interacts with P2Y_1_ receptors expressed in astrocytes, to prevent the ADPβS-induced astroglial proliferation.

**FIGURE 6:**
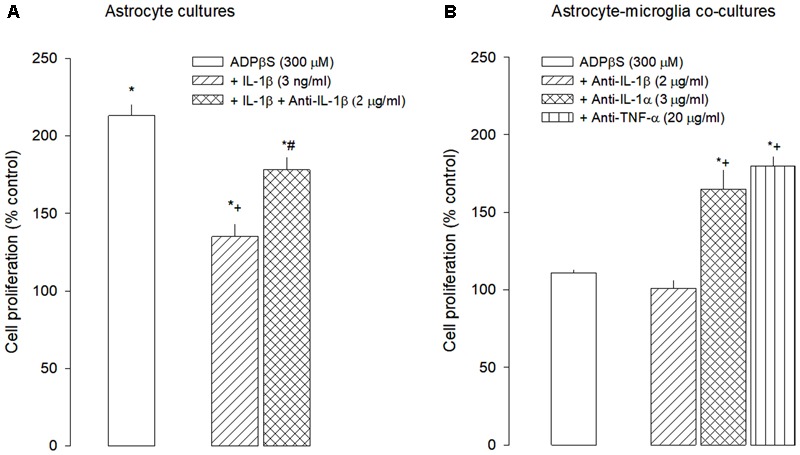
Effect of IL-1β and anti-IL-1β antibody on proliferation induced by ADPβS in astrocyte cultures **(A)** and effect of antibodies anti-IL-1β, anti-IL-1α, and anti-TNF-α in proliferation induced by ADPβS on astrocyte-microglia co-cultures **(B)**. **(A)** Astrocyte cultures were incubated with ADPβS alone or in combination with IL-1β or IL-1β plus anti-IL-1β antibody for 48 h. **(B)** Astrocyte-microglia co-cultures were incubated with ADPβS or with anti-ILs antibodies alone or with ADPβS plus anti-ILs antibodies for 48 h. Anti-ILs antibodies, when present were added at the same time as ADPβS or solvent. *Methyl*-[^3^H]-thymidine (1 μCi/mL) was added in the last 24 h and cell proliferation was estimated by *methyl*-[^3^H]-thymidine incorporation and expressed in percentage of control. Values are means ± SEM from 4 to 5 different cultures. ^∗^*P* < 0.05, significant differences from the respective control; ^+^*P* < 0.05, significant differences from ADPβS alone and ^#^*P* < 0.05, significant differences from ADPβS plus the anti-IL-I1β antibody.

In agreement with this hypothesis, in astrocyte cultures, IL-1β attenuated ADPβS-induced astroglial proliferation, and this effect was prevented by the anti-IL-1β antibody (**Figure 6A**), supporting the view that IL-1β has conditions to exert such role. Additionally, when tested alone, the anti-IL-1β antibody had no effect, suggesting that there is no significant tonic release of IL-1β by astrocytes or by the small percentage of contaminating microglia.

Considering the hypothesis that IL-1β could be the soluble messenger produced by microglia, responsible for preventing ADPβS-induced astroglial proliferation, the anti-IL-1β antibody was tested in co-cultures to investigate whether, after IL-1β neutralization, ADPβS was able to induce astroglial proliferation. In co-cultures, when tested alone, the anti-IL-1β antibody induced cell proliferation up to 142 ± 4 (*n* = 5; *P* < 0.05), suggesting the occurrence of a basal release of IL-1β by microglia exerting a tonic inhibition of astroglial proliferation. However, the anti-IL-1β antibody was unable to restore the ADPβS proliferative effect (**Figure 6B**), indicating that there is no additional significant release of IL-1β, when ADPβS activates microglial P2Y receptors (not shown).

Following the same hypothesis, and considering the contribution of activated microglia to the release of other interleukins, such as IL-1α and TNF-α (Liu et al., 2017; Liddelow et al., 2017), that have been shown to change de reactivity of astrocytes (Liddelow et al., 2017), the interaction between ADPβS and the antibodies anti-IL-1α and anti-TNF-α was tested in co-cultures. Unlike the anti-IL-1β antibody, both the anti-IL-1α and the anti-TNF-α antibodies had no effect when tested alone, but restored the ADPβS-proliferative effect to levels close to those observed in astrocyte cultures (**Figure 6B**). These results suggest that activation of microglia P2Y_12,13_ receptors by ADPβS may induce the release of IL-1α and TNF-α that control astroglial proliferation.

## Discussion

Nucleotides, such as ATP and ADP are massively present in the extracellular medium during brain lesion, and were shown to activate P2Y receptors, modulating astroglial proliferation through mechanisms that involve communication between microglia and astrocytes (Quintas et al., 2011b). ADPβS is a stable ADP analog, selective for P2Y_1_, P2Y_12_, and P2Y_13_ receptor subtypes, and was shown to cause cell proliferation in astrocyte cultures, an effect mediated by P2Y_1_ and P2Y_12_ receptors (Quintas et al., 2011a). The ADPβS-induced astroglial proliferation was abolished when the percentage of microglia cells increased to about 10–13% microglia (Quintas et al., 2011b). In these previous studies, it was excluded the possibility that a lower expression of P2Y_1_ and/or P2Y_12_ receptors, or a preferential microglial localization of these P2Y receptor subtypes could explain the absence of ADPβS-induced proliferative effect observed in co-cultures. It was also demonstrated that the presence of microglia did not influence metabolic degradation of ADPβS. Therefore, an activation of microglia P2Y receptors by ADPβS, was seen as the more solid explanation for the lack of ADPβS proliferative effect observed in co-cultures. However, the microglial P2Y receptor subtype(s) involved in the modulation of astroglial proliferation remained to be identified.

In the present study, it was confirmed that, in astrocyte cultures, ADPβS induces cell proliferation. Despite the minor contamination of astrocyte cultures with microglia, we have demonstrated, by BrdU incorporation, that astrocytes are the main proliferating cells. ADPβS proliferative effect in these cultures is triggered through activation of P2Y_1_ and P2Y_12_ receptor subtypes. The ADPβS proliferative effect was attenuated by the selective P2Y_1_ receptor antagonist, MRS 2500 (1 μM; Houston et al., 2006) and by the selective P2Y_12_ receptor antagonist, AR-C66096 (0.1 μM; Humphries et al., 1994). Interestingly, no additive effect was observed when both antagonists were tested simultaneously. A possible explanation is that, although they are coupled to different transduction mechanisms, they may convey in a common pathway. In fact, P2Y_1_ receptors, are coupled to G(q) proteins and mediate astroglial proliferation through activation of phospholipase (PLC)-protein kinase C (PKC)-extracellular signal-regulated kinase 1/2 (ERK1/2) pathway (Neary et al., 2003; Quintas et al., 2011a), whereas P2Y_12_ receptor activation may lead to G(i) βγ-dependent PLC-PKC-ERK1/2 pathway, as seen in 1321N1 human astrocytoma cells (Mamedova et al., 2006) or to a PLC-independent activation of PKC, as seems to occur in glioma C6 cells (Grobben et al., 2001; Van Kolen and Slegers, 2006). Therefore, P2Y_1_ and P2Y_12_ receptors subtypes, despite being coupled to different G proteins, may activate converging pathways, leading to ERK1/2 activation and cell proliferation. The results obtained in astrocyte cultures further demonstrate that P2Y_13_ receptors are not involved in astroglial proliferation, because the antagonist of P2Y_13_ receptor MRS 2211 (3 μM; Kim et al., 2005) did not change the ADPβS-induced astroglial proliferation.

In co-cultures, the results obtained with ADPβS on proliferation contrast with those observed in astrocyte cultures. In co-cultures, ADPβS failed to induce astroglial proliferation. However, the proliferative effect of ADPβS was restored, to levels similar to those observed in astrocyte cultures, when P2Y_13_ receptors were blocked with MRS 2211, and partially recovered when P2Y_12_ receptors were blocked with AR-C66096.

P2Y_12_ receptors have been shown to be expressed either by microglia (Haynes et al., 2006; Ohsawa et al., 2007; De Simone et al., 2010) or by astrocytes (Fumagalli et al., 2003, 2004; Carrasquero et al., 2005; Amadio et al., 2010). Additionally, our previous studies demonstrated, by western blot and by immunocytochemical assays, that P2Y_12_ receptors are expressed in both astrocytes cultures and co-cultures, by astrocytes and by microglial cells, with no preferential cellular localization (Quintas et al., 2011b). In this study, P2Y_12_ receptors expression in astrocytes and microglia was further supported by quantification of P2Y_12_ transcripts.

Concerning to the P2Y_13_ receptors, they were expressed at low levels either in astrocyte cultures or co-cultures, which is in agreement with its lower mRNA expression in astrocytes. P2Y_13_ receptors have been shown to be expressed in astrocytes of several brain regions, inducing [Ca^2+^]_i_ increase (Fumagalli et al., 2004; Carrasquero et al., 2009; Fischer et al., 2009), which suggest a possible role for this receptor subtype in the modulation of astrogliosis. In the present work, P2Y_13_ receptors were shown to be residually expressed by astrocytes, but do not directly modulate of astroglial proliferation. Results obtained by immunocytochemistry showed that they were preferentially expressed by microglia, an observation in line with previous studies that demonstrated the expression of P2Y_13_ receptors in microglia from the whole brain (Crain et al., 2009) and spinal cord, where they elicit [Ca^2+^]_i_ increase (Zeng et al., 2014) and the release of several pro-inflammatory cytokines, such as IL-1β, IL-6 and TNF-α (Liu et al., 2017).

From the pharmacological and molecular biology results, it may be concluded that astrocyte P2Y_13_ receptors do not directly mediate astroglial proliferation, but rather act indirectly, through microglia, to regulate P2Y-mediated astroglial proliferation. The P2Y_12_ receptors seem to cooperate with P2Y_13_ receptors to restrain astroglial proliferation in co-cultures.

In co-cultures, P2Y_12_ and P2Y_13_ receptors also do not cause additive effects in preventing the ADPβS-induced astroglial proliferation. Likely, because P2Y_12_ and P2Y_13_ share the same transduction pathways. Both receptor subtypes are coupled to G(i) proteins (Marteau et al., 2003) and/or to an increase in [Ca^2+^]_i_, as described in microglial cells (Bianco et al., 2005).

We have previously shown that conditioned medium of microglia treated with ADPβS prevented the proliferative effect mediated by P2Y_1,12_ receptors in astrocyte cultures, supporting the view that, in co-cultures, activation of microglia P2Y receptors induced the release of diffusible paracrine mediator(s) responsible for the inhibitory influence in the ADPβS-induced astroglial proliferation (Quintas et al., 2011b).

A recent report has shown that microglial cytokines, such as IL-1β, TNF-α and IL-6, transform astrocytes into a neuroprotective phenotype, involving downregulation of P2Y_1_ receptors (Shinozaki et al., 2017). We have also shown that, the presence of microglia was associated with a loss of P2Y_1_ receptor function, but without changing the expression levels (Quintas et al., 2011b). In line with our observations, it was seen a correlation between the presence of the pro-inflammatory IL-1β and the loss of P2Y_1_ receptors function in astrocytes, without modifying its expression levels, possibly due to protein-protein interactions (Scemes, 2008). Thus, it was considered the hypothesis that microglial P2Y_12,13_ receptors could induce the release of IL-1β which, acting on astrocytes, would prevent the P2Y_1,12_-mediated proliferation. According to this hypothesis, IL-1β inhibited ADPβS-induced proliferation in astrocytes cultures, an effect prevented by the anti-IL-1β antibody. In co-cultures, but not in astrocyte cultures, the anti-IL-1β antibody, induced astroglial proliferation, suggesting that there is a microglial basal release of IL-1β that tonically inhibits astroglial proliferation. However, ADPβS was still unable to induce astroglial proliferation in the presence of anti-IL-1β antibody, excluding any significant contribution of IL-1β to the microglial P2Y_12,13_ receptor-mediated inhibition of astroglial proliferation.

Another recent study, indicated that activated microglia release IL-1α, TNF-α and the complement factor Cq1, which are involved in phenotypical changes in astrocytes that may lead to a less proliferative and more aggressive profile to neurons (Liddelow et al., 2017). In line with this study it was hypothesized that in co-cultures, ADPβS-activated microglia could release IL-1α, TNF-α, and that these interleukins could be paracrine mediators involved in the inhibition of ADPβS-mediated astroglial proliferation. In co-cultures, both anti-IL-1α and anti-TNF-α antibodies restored, almost completely, the ADPβS proliferative effect, without causing significant effects on basal proliferation, supporting the conclusion that activation of microglia P2Y_12,13_ receptors by ADPβS may induce the release of IL-1α and TNF-α that control P2Y_1,12_ astroglial proliferation.

Taken together, the present results evidence the existence of two distinct pair of receptors controlling astroglial proliferation induced by extracellular purine nucleotides: P2Y_1_ and P2Y_12_ receptors, present in astrocytes and causing proliferation, and P2Y_12_ and P2Y_13_ receptors, present in microglia, causing a suppression of astroglial proliferation due to the release of soluble messenger(s), on a paracrine mode of communication that is independent of IL-1β, but seems to involve the release of IL-1α and TNF-α.

This purinergic interaction between microglia and astrocytes may be relevant under physiopathological conditions, during the initial phase of the inflammatory response, when there is cell death and inflammation, recruitment of microglia and other inflammatory and immune cells to remove cell debris (Burda and Sofroniew, 2014). Nucleotides are released to the lesion core at these early stages of CNS insult and may coordinate multicellular responses, activating purinergic receptors in astrocytes, microglia and surrounding cells (Buffo et al., 2010). Adenine nucleotides have been shown to induce astroglial proliferation mediated by P2Y_1,12_ receptors (Franke et al., 2004; Quintas et al., 2011a) and would also act as chemotactic signals through activation of P2Y_12_ receptors, causing microglia mobilization to reach the damaged site and to modulate the inflammatory response (Davalos et al., 2005). Here, we demonstrate that activation of microglial P2Y_12,13_ receptors control P2Y_1,12_ receptor-mediated astroglial proliferation. With the arrival of microglia to the lesion core, microglial P2Y_12,13_ receptors would silence P2Y_1,12_ receptor-mediated astroglial proliferation, by the release of paracrine mediators, delaying the formation of the astrocytic scar and, therefore, keeping open the path for more immune cells infiltration. As inflammation is resolved, microglia will change their phenotype, releasing astrocytes from the P2Y_12,13_ proliferative brake, and pave the way for a full remodeling and repair.

## Ethics Statement

Animal handling and experiments were conducted according to the guidelines of the Directive 2010/63/EU of the European Parliament and the Council of the European Union. Ethical commission of the animal house, called Organismo Responsável pelo Bem-Estar Animal (ORBEA), which in English corresponds to “Commission responsible for Animal Welfare,” approved this study. Additionally, a qualified veterinary supervised the most critical part, which consisted in the euthanasia of newborn rats.

## Author Contributions

GQ supervised the entire work, prepared the cell cultures, and performed the experiments of DNA synthesis. CQ prepared the cell cultures and performed the experiments of immunofluorescence and molecular biology. GQ and CQ conceived and designed the experiments and analyzed the data. JG and NV analyzed the data and critically revised the manuscript. All authors discussed the results and contributed to manuscript writing.

## Conflict of Interest Statement

The authors declare that the research was conducted in the absence of any commercial or financial relationships that could be construed as a potential conflict of interest.
