# Supplementary material for: Microglia P2Y13 Receptors Prevent Astrocyte Proliferation Mediated by P2Y1 Receptors
Source: Front Pharmacol. 2018 May 3;9:418. doi: 10.3389/fphar.2018.00418 (PMC5943495; doi:10.3389/fphar.2018.00418)
Supplement: Supplementary file 1 [file Image_1.pdf]

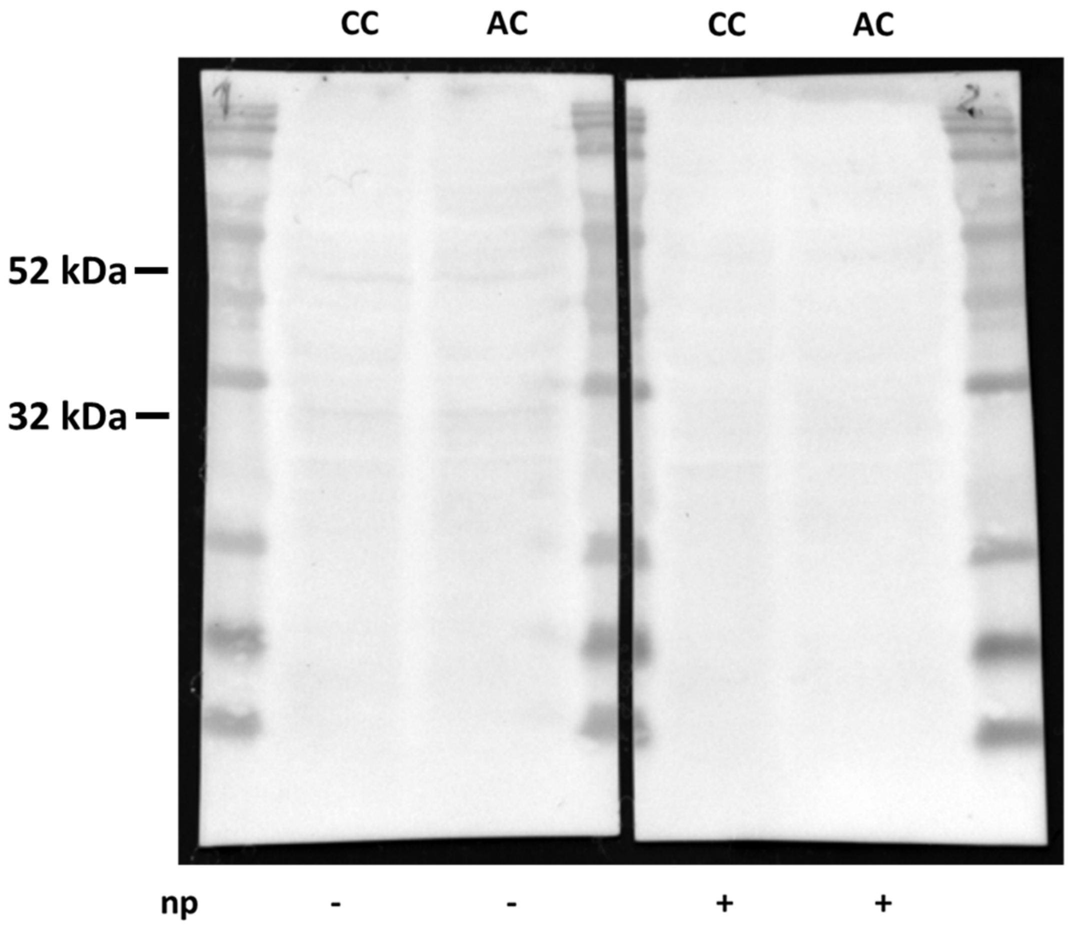

Supplementary Fig. 1 Whole blot of $P2Y_{13}$ receptors ($P2Y_{13}R$) expression in astrocyte-microglia co-cultures (CC) and astrocyte cultures (AC). $P2Y_{13}R$ expression were obtained from whole cell lysates. Two immunoreactive bands of 32 and 52 kDa specifically reacted with rabbit anti-$P2Y_{13}$ antibody. These bands were absent in the presence of the respective neutralising peptide (np).

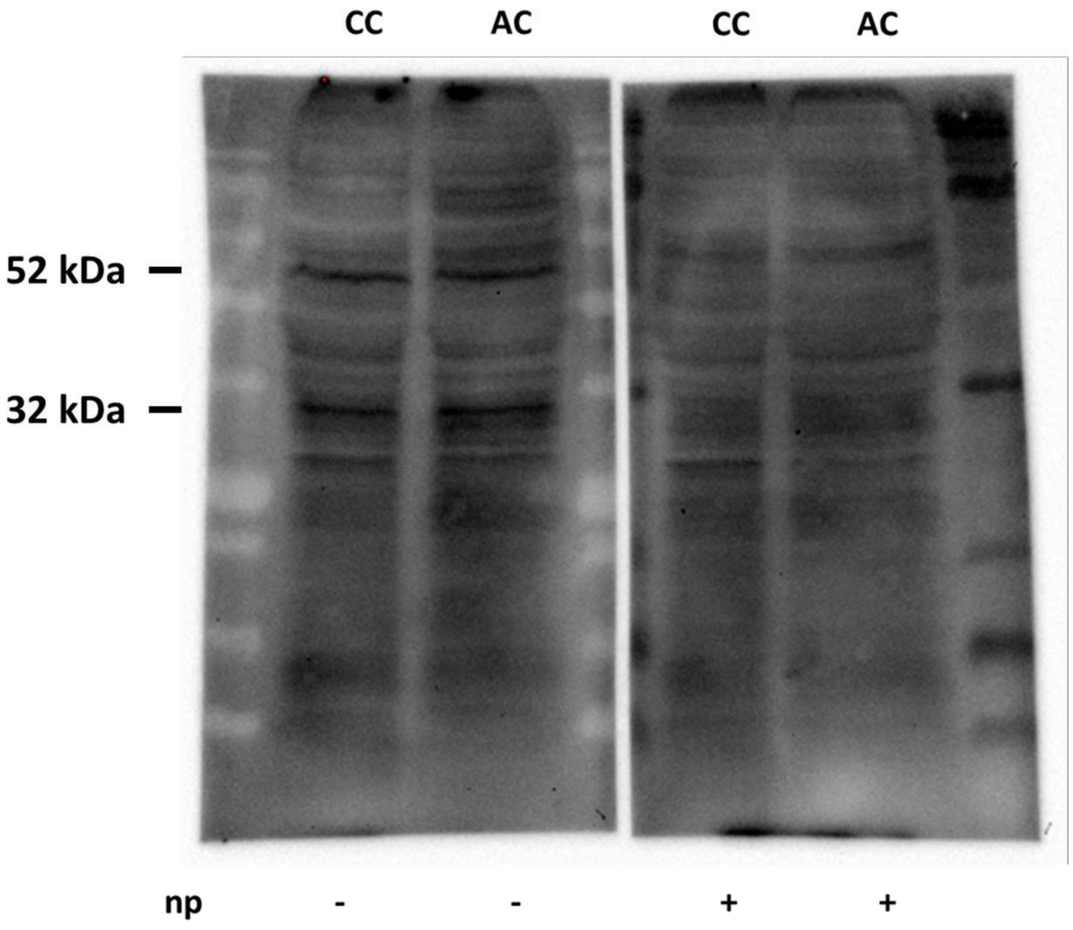

Supplementary Fig. 2 Whole overexposed blot of $P2Y_{13}$ receptors ($P2Y_{13}R$) expression in astrocyte-microglia co-cultures (CC) and astrocyte cultures (AC). $P2Y_{13}R$ expression were obtained from whole cell lysates. Two immunoreactive bands of 32 and 52 kDa specifically reacted with rabbit anti-$P2Y_{13}$ antibody. These bands were absent in the presence of the respective neutralising peptide (np).
